# Comparative Evaluation of the Antimicrobial Effect of Mangosteen, Triphala, Chitosan, and 2% Chlorhexidine on Mono- and Dual-Species Biofilms of – and *Candida albicans*: An *in Vitro* Study

**DOI:** 10.14744/eej.2021.70783

**Published:** 2022-03-10

**Authors:** Vinoo Subramanaiam RAMACHANDRAN, Mensudar RATHAKRISHNAN, Malathy Balaraman RAVINDRRAN, Venkatesh ALAGARSAMY

**Affiliations:** From the Department of Conservative Dentistry and Endodontics (V.S.R. 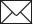 vinoomds@yahoo.com), Bharath Institute of Higher Education and Research, Tamilnadu, India; Department of Conservative Dentistry and Endodontics (M.R., V.A.) Sree Balaji Dental College and Hospital, Tamilnadu, India; Department of Microbiology (M.B.R.), Sathyabama Dental College and Hospital, Tamilnadu, India

**Keywords:** *Candida albicans* biofilm, chitosan, *Enterococcus faecalis* biofilm, mangosteen, triphala

## Abstract

**Objective:**

The purpose of this *in vitro* study was to compare the antimicrobial effectiveness of 2% chlorhexidine gel, 0.5% chitosan, ethanol extract of mangosteen pericarp, and Triphala used as intracanal medicaments against *Enterococcus faecalis* and *Candida albicans* in mono- and dual-species biofilms.

**Methods:**

Bioactive components in the ethanol extract of mangosteen pericarp and Triphala were evaluated by gas chromatography-mass spectrometry (GCMS). Quantitative assessment of the biofilm formations of *E. faecalis* and *C. albicans* and as a dual-species in the presence of test medicaments was carried out using a crystal violet (CV) assay in a microtiter plate. Following this, 246 single-rooted premolar teeth were collected, and root specimens were prepared. *C. albicans* and *E. faecalis* mono- and dual-species biofilms were grown in the root specimens. At the end of 21 days, the samples were divided into five groups and subjected to different types of medicaments: Control group- distilled water; Chlorhexidine group- 2% chlorhexidine gel; Chitosan group- 0.5% chitosan; Mangosteen group- ethanol extract of mangosteen pericarp; and Triphala group- ethanol extract of Triphala. Colony-forming units (CFUs) were assessed on the first and fifth day after medicament placement.

**Results:**

Microbial population reduction was measured by one-way analysis of variance, followed by post-hoc Tukey’s multiple comparison test (P<0.05). Chlorhexidine showed maximum log reduction in CFUs of microorganisms, followed by chitosan, which showed a similar log reduction (P>0.05) for both mono- and dual-species biofilms. However, in the mangosteen and Triphala extract groups, the CFU/mL for dual-species on both days did not have a significant reduction in count (P<0.05) when compared to chlorhexidine and chitosan.

**Conclusion:**

Chlorhexidine showed maximum antimicrobial activity, followed by chitosan, on both mono- and dual-species biofilms. Mangosteen and Triphala had good antimicrobial action on the mono-species biofilm.

**HIGHLIGHTS**

• This study evaluated the antimicrobial efficacy of 2% chlorhexidine gel, 0.5% chitosan, and ethanol extracts of mangosteen pericarp and Triphala used as intracanal medicaments on mono- and dual-species biofilms.
• 0.5% chitosan showed antimicrobial efficacy similar to 2% chlorhexidine. Mangosteen and Triphala had good antimicrobial action on mono-species biofilm.
• The protective role of extracellular polysaccharide structures and microbial interactions in dual-species biofilms can cause resistance to medicament action.
• Natural extracts can serve as a source for discovering novel intracanal medicaments from plant sources.

## INTRODUCTION

Microbial biofilms are the major cause of primary and secondary root canal infections, and the success of endodontic treatment relies on its effective eradication. Bacteria, existing as organized biofilm communities, are highly resistant to chemomechanical debridement procedures. The anatomical complexities of the root canal system may pose significant challenges for proper cleaning, shaping, and disinfection (1). Intracanal medicament can be used to reduce the bacterial load and also they aid in proper canal disinfection and achieve a biologically acceptable environment before canal obturation (2, 3). *Enterococcus faecalis* is the most frequently isolated species in failed root canal-treated teeth (4). The yeast *Candida albicans* is also frequently co-isolated from polymicrobial biofilm infections and can interact with different bacteria in various ways and enhance antimicrobial resistance (5). The yeast cells can form long hyphal elements, thereby creating a physical structure to support the formation of bacterial biofilm (6). *E. faecalis* is found to have more resistance to starvation in the presence of *C. albicans* (7). A study conducted by Ozok et al. (8) showed that dual-species biofilms were more resistant to endodontic irrigants compared to mono-species biofilms. This enhanced antimicrobial resistance is related to the difference in the composition of the extracellular polysaccharide (EPS) in multispecies biofilms compared to mono-species biofilm.

Cytotoxicity and other harmful side effects of commonly used antimicrobial agents have necessitated the need for alternative agents. Herbal alternatives have recently gained popularity as a result of their antibacterial potency, low toxicity, ease of availability, and cost-effectiveness. Mangosteen fruit peel contains abundant amounts of xanthones and other bioactive compounds, including flavonoids, tannins, and anthocyanins. Studies have also shown that mangosteen peel extract effectively inhibits *Streptococcus mutans* and *Porphyromonas gingivalis* biofilms (9). Triphala consists of three medicinal plants, *Terminalia chebula, Terminalia belerica*, and *Emblica officinalis*, which have good antimicrobial action (10). Chitosan is a biodegradable, natural, nontoxic polysaccharide having broad-spectrum antimicrobial activity against Gram-positive bacteria, Gram-negative bacteria, and fungi (11). Chlorhexidine has broad-spectrum antimicrobial activity and is used as a root canal irrigant and medicament. However, chlorhexidine lacks tissue dissolving properties, and at higher concentrations, it induces necrosis, and the lower concentrations are associated with apoptosis (12). In the view of enhanced antimicrobial resistance of dual-species biofilm compared to mono-species biofilm, this study analyses a dual-species model consisting of *E. faecalis* and *C. albicans*. Considering the advantages of using natural extracts, the present *in vitro* study was undertaken to evaluate the antimicrobial properties of mangosteen, Triphala, and chitosan when used as intracanal medicaments during endodontic treatment, compared with chlorhexidine on mono-species and dual-species interkingdom biofilms formed on tooth root specimens.

## MATERIALS AND METHODS

### Medicament preparation

Ethanol was used as a solvent to prepare the mangosteen and Triphala extracts. Dried mangosteen pericarp powder and Triphala powder (IMCOPS, Chennai, India) were used for medicament extraction. Mangosteen pericarp and Triphala powder 100 g each were added to 50 mL of absolute ethanol, and the extract was prepared using a Soxhlet apparatus. The alcohol part was removed to get approximately 25 mL of the concentrated extract, and the extract was stored in an airtight amber-coloured container for further use. Using this concentrated extract, various volumes of alcoholic mangosteen and Triphala extracts measuring 50 mg/mL, 100 mg/mL, 200 mg/mL, 300 mg/mL, 400 mg/mL, and 500 mg/mL were used for identifying concentrations with maximum antimicrobial action, indicating minimum inhibitory concentration (MIC). Chitosan of low molecular weight was obtained from Panvo Organics Limited (India), and 0.5 g of chitosan was diluted in 100 mL of 1% acetic acid to get a concentration of 0.5% chitosan.

### Gas chromatography-mass spectrometry (GC-MS) analysis

The phytochemical constituents in the ethanol extracts of mangosteen and Triphala were identified using GC-MS analysis (Clarus SQ 8C Gas Chromatography-Mass Spectrometer from Perkin Elmer). Ethanol fractions were prepared from previously obtained concentrated extracts. The extract was again dissolved in ethanol and passed through a 0.45 μm filter before injecting into the GC-MS. The results were interpreted using the database of the National Institute of Standards and Technology (NIST14; https://chemdata.nist.gov/dokuwiki/doku.php?id=chemdata:start).

### Microbial strains used

*E. faecalis* ATCC 29212 and *C. albicans* ATCC 24433 (HiMedia, India) were used in the study. *E. faecalis* and *C. albicans* were grown in tryptone soya broth (HiMedia, India) for 24 hours at 37°C, and the cell load was adjusted to 1.5×10^7^ CFU/mL using McFarland’s standard.

### Antimicrobial activity of the extract and chitosan by well diffusion

The antimicrobial compound activity was measured by the agar well diffusion method. A microbial lawn was prepared on tryptone soya agar (TSA) by spreading 100 µL (1.5×10^7^ CFU/mL of *E. faecalis*/*C. albicans*. Then, a well with a diameter of 8 mm was punched aseptically with a sterile cork borer. Various concentrations of the extracts (50 mg/mL, 100 mg/mL, 200 mg/mL, 300 mg/mL, 400 mg/mL, and 500 mg/mL) were tested. Exactly 20 µL of the concentration was loaded into each well separately; the plates were incubated under 37 C for 24 hours, and the zone of inhibition was measured (13). Each medicament concentration was replicated thrice, and ten Petri dishes were maintained for each replication. The least extract concentration that showed the maximum zone of inhibition was taken as the minimal inhibitory concentration. The negative control (only solvent) and positive control (2% chlorhexidine) were also included.

### Effect of medicaments on biofilm formation in the culture medium

The minimum inhibitory concentration of the extract was used to test its efficacy against biofilm formation. Quantitative assessment of the biofilm formations of *E. faecalis* and *C. albicans* and as a dual-species was carried out using the crystal violet (CV) assay in a microtiter plate. Briefly, fresh tryptone soya broth (100 μL) having MIC of the medicament was seeded with 10 μL of *E. faecalis* or *C. albicans* and each 5 μL of *E. faecalis* plus *C. albicans* in a 96-well microtiter plate. The plates were incubated at 37°C for 24 hours. After incubation, the planktonic cells were aspirated, and the wells were washed with sterile water to remove the free-floating bacteria. The adhering biofilm was fixed by heating and then stained with 0.1% crystal violet. 

The wells were washed with sterile water and dried. The amount of biofilm formed was quantified by the addition of 200 μL of 95% ethanol/acetic acid mixture (1:1), and the optical density (OD) of the resulting solution was measured at 595 nm in a double beam spectrophotometer (Shimadzu, Japan). Background absorbance was compensated for by subtracting the OD of the sterile broth (negative control). The experiment was carried out in triplicate, and the mean OD was considered.

### Assay of Extracellular Polysaccharide produced by test strains

The microorganisms were tested for their ability to produce extracellular polysaccharides in the presence of the test medicaments. EPSs were extracted from a 3-day-old culture, grown in tryptone soya broth amended with MIC of the test medicaments at 37°C. Two mL of the culture was centrifuged at 10,000 rpm for 10 minutes, and 1 mL of the supernatant was collected, to which 2 mL of 90% ethanol was added and incubated at 20°C for 24 hours. This mixture was then centrifuged at 10,000 rpm for 15 minutes, and the precipitate was dissolved in 2 mL of water. Then 200 μL of 5% phenol and 1 mL of 93% sulphuric acid were added and kept at room temperature for 10 minutes to form a yellow colour, indicating positive EPS production. The absorbance was recorded at 490 nm using a spectrophotometer. The calibration curve was prepared using the standard stock solution of 10 ppm glucose at different concentrations. The EPS production was expressed in the release of reducing sugars as μg/mL.

### Effect of the medicaments on biofilm formation on the tooth model

#### Preparation of the root samples and biofilm formation on the tooth substrate

Sample size calculation was carried out using G*Power 3.1 software. Ethical approval was obtained from the institutional ethical board (SBDCH/IEC/12/2020/22). A total of 246 noncarious mature human single-rooted teeth, freshly extracted for orthodontic purposes, were selected for the study. The teeth were cleaned and a rotary diamond disk was used to decoronate the teeth 4 to 5 mm below the cementoenamel junction to obtain a standardised root length of 12 mm. The root canals were then instrumented using rotary instruments, and canals were prepared to apical size 30 using a ProTaper F3 file (ProTaper, Dentsply Maillefer, Switzerland) combined with 2.5% NaOCl irrigation. Organic and inorganic debris were removed by treating the root sample with 17% ethylenediaminetetraacetic acid for 5 minutes, followed by 3% sodium hypochlorite for 5 minutes (9, 10). Following this, six root samples alone were chosen randomly from the prepared 246 root blocks and were sectioned vertically along the mid-sagittal plane into two halves to be used for scanning electron microscopy (SEM) evaluation. The concave tooth surfaces of the vertically split teeth were then minimally ground to achieve a flat surface to allow proper microbial biofilm formation. The purpose of this SEM analysis of the six selected tooth samples was to confirm biofilm formation following microbial inoculation. The remaining 240 samples were used for CFU analysis after medicament treatment.

All the prepared root samples (n=246) were subjected to sterilisation in an autoclave at 121°C for 15 minutes. This was followed by a second cycle of sterilisation during which the blocks were immersed in 1 mL of tryptone soya broth in individual microcentrifuge tubes for better penetration of the broth into the dentinal tubules (11). Then, 10 µL of *E. faecalis* (10^7^ CFU/mL) or *C. albicans* (10^7^ CFU/mL) and, for dual-species, 5 µL of *E. faecalis* (10^7^ CFU/mL) plus 5 µL *C. albicans* (10^7^ CFU/mL) were inoculated and incubated at 37°C in a shaking incubator for 21 days. The fresh broth was changed every other day. Finally, the purity of the culture was checked by inoculating 10 µL of the broth from the incubated root specimens from each tube onto the tryptone soya agar. All procedures were carried out inside a laminar airflow cabinet.

#### SEM evaluation

At the end of 21 days, six teeth specimens that were sectioned vertically were analysed under a scanning electron microscope for biofilm formation confirmation. The tooth samples were fixed with 2.5% glutaraldehyde (v/v) for 1 hour at room temperature, washed with buffer solution, followed by dehydration of the fixed specimens using 10% ethanol for 5 min. Then the dentine blocks were sputter-coated with gold nanoparticles to prevent structural damage when subjected to high-voltage electron beam and vacuum. Finally, the dentine blocks were visualised through SEM (Quanta 250, FEI, The Netherlands).

### Group assignment, medicament placement, and root dentine sampling

After 21 days, the contaminated broth was removed from the canals with a sterile syringe. The canals were washed with saline to remove planktonic bacteria and blotted dry with sterile paper points. The remaining 240 specimens (excluding the six which were taken for SEM analysis) were randomly divided into four experimental groups and one control, i.e., distilled water group (n=48 in each group). The groups were further subdivided, which is represented in the flowchart. Methylcellulose was used as a thickening agent for the medicaments. Under aseptic conditions, the canals of the experimental groups were injected with the medicaments until they were full. The control teeth were filled with distilled water. The dentine blocks’ opening was sealed with paraffin wax. The specimens were incubated at 37°C.

The following medications were examined:

Control group-distilled water control; 

Chlorhexidine group-2% chlorhexidine gel; 

Chitosan group-0.5% chitosan mixed with methylcellulose; 

Mangosteen group-mangosteen extract mixed with methylcellulose;

Triphala group-Triphala extract mixed with methylcellulose.

**Figure 1.**
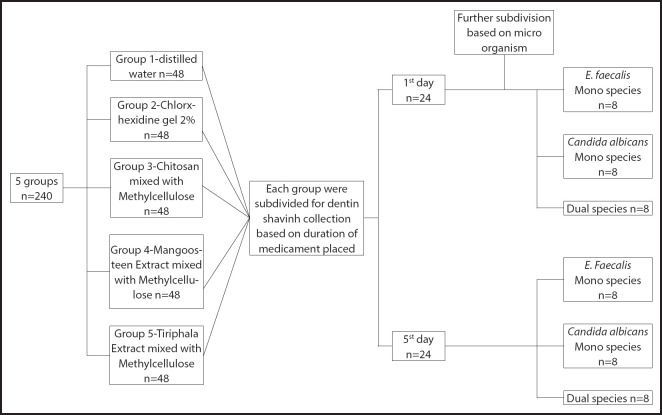
Grouping of samples

The groups are further subgrouped as shown in the flowchart (Fig. 1).

Samples of the dentinal shavings were collected from all groups on the first and fifth day after exposure to medicaments. For this, the dentine blocks were removed from the microcentrifuge tube, and the medicaments were removed with a sterile syringe; the canals were rinsed with 10 mL of sterile saline and dried with sterile paper points. Dentinal shavings were collected using a Peeso Reamer (ManiR, Utsunomiya, Tochigi, Japan) size no. 4 equivalent to 1.3 mm diameter using a low-speed handpiece. Only one stroke was made to standardise the volume of dentinal debris collected.

### Antimicrobial assessment of medicaments on root canal dentine

The collected dentinal shavings were transferred into a microcentrifuge tube (Axygen, NY, USA) containing one mL sterile tryptone soya broth. Next, a sterile microtip was used to take 100 μL of the broth containing dentinal shavings and transferred to another tube containing 900 μL sterile tryptone soya broth. The content of each tube was shaken and serially diluted from 10^-1^ to 10^-6^. Subsequently, 300 μL of the diluted dentinal shavings was spread uniformly on triplicated TSA plates using an L-shaped glass rod and incubated at 37°C for 24 hours. Then the colonies were counted, and readings were tabulated. The efficiency of the disinfectant activity on the biofilm formed on tooth substrate was calculated through the reduction of CFUs between the untreated distilled water group and the medicament group. 

### Statistical analysis

The inhibition of biofilm formation and CFUs were analysed statistically using IBM SPSS software v.22.0. The CFU data were statistically analysed with a one-way analysis of variance, followed by post-hoc Tukey’s multiple comparison test of means to check the difference in reduction of microbial count (P<0.05). The independent sample paired t-test was used to check for differences in growth at different time intervals.

## RESULTS

### Gas chromatography-mass spectrometry (GC-MS) analysis

Bioactive compounds present in the ethanol extract of mangosteen and Triphala are presented in Tables 1 and 2.

**TABLE 1.** Gas chromatography-mass spectrometry (GC-MS) analysis of the ethanolic extract of mangosteen

Retention time (Minutes)	Area (%)	Name of compound
4.269	0.814	Propane, 1,1-diethoxy-2-methyl-
5.134	0.397	Propane, 1,1,3-triethoxy-
7.350	0.554	2-Methoxy-4-vinylphenol
9.406	0.508	2-Cyclohexen-1-one, 3-(hydroxymethyl)-6-(1-methylethyl)-
9.471	0.675	Benzene, 1-(1,5-dimethyl-4-hexenyl)-4-methyl-
12.502	0.423	alpha-Atlantone
12.622	1.214	aR-Turmerone
12.782	0.705	Quinine
13.387	0.311	2-Methyl-6-(4-methylenecyclohex-2-en-1-yl)hept-2-en-4-one
15.633	0.907	3-Buten-2-one, 4-(4-hydroxy-3-methoxyphenyl)-
18.629	4.430	n-Hexadecanoic acid
21.716	5.309	9,12-Octadecadienoic acid (Z,Z)-
21.851	11.029	9-Octadecenoic acid, (E)-
22.306	2.796	Octadecanoic acid
25.702	0.425	7,8-Epoxylanostan-11-ol, 3-acetoxy-
27.978	4.596	Ergost-7-en-3-ol
28.248	0.645	Diisooctyl phthalate
28.894	6.477	Tetratetracontane
29.189	4.887	Stigmast-7-en-3-ol,

**TABLE 2.** Gas chromatography-mass spectrometry (GC-MS) analysis of the ethanolic extract of Triphala

Retention time (Minutes)	Area (%)	Name of compound
3.874	2.729	Propane, 1,1,3-triethoxy-
5.519	0.434	2(3H)-Furanone, 5-acetyldihydro-
6.295	0.395	5-Hydroxymethylfurfural
6.615	0.603	Butanedioic acid, hydroxy-, diethyl ester
8.335	1.404	1,2,3-Benzenetriol
8.401	6.448	Pyrazole-5-carboxylic acid, 3-methyl-
9.051	1.217	N,N,Diacetyl-Lys-DAla-DAla
9.371	1.606	1-Isobutoxycarbonyl-4(5)-methylimidazole
9.776	1.869	2,4-Di-tert-butylphenol
10.236	0.223	1,2,4-Cyclopentanetrione, 3-(1-methylbutyl)-
10.376	0.286	1,7-Octadien-3-ol, acetate
18.620	5.773	n-Hexadecanoic acid
19.200	0.358	Hexadecanoic acid, ethyl ester
20.510	0.590	Beta-Sitosterol
21.341	0.222	Cedilla–Sitosterol
21.791	10.680	9,12-Octadecadienoic acid (Z,Z)-
21.936	10.884	9-Octadecenoic acid, (E)-
24.492	0.811	Glycidyl palmitate
25.542	0.348	Stigmasterol
25.682	0.552	Eicosanoic acid
27.383	1.521	9,12-Octadecadienoic acid (Z,Z)-, 2-hydroxy-1-(hydroxymethyl) ethyl ester
27.488	2.154	Glycidyl oleate
27.898	0.801	Octadecanoic acid, 2-hydroxy-1,3-propanediyl ester
28.013	1.768	Hexadecanoic acid, 2-hydroxy-1-(hydroxymethyl)ethyl ester
28.794	10.322	2,5-Cyclohexadien-1-one, 4-[3,5-bis(1,1-dimethylethyl)-4-oxo-2,5- cyclohexadien-1-ylidene]-2,6-bis(1,1-dimethylethyl)-
28.924	10.562	Cromolyn
29.584	9.261	Lupeol

### Antimicrobial activity by agar well diffusion method

The antimicrobial assessment by agar well diffusion assay showed that the mean zone of inhibition (values expressed as mean±SD with 95% confidence interval) of *E. faecalis* and *C. albicans* was maximum for 2% chlorhexidine (29±0.4 mm and 26±0.1 mm, respectively), followed by 0.5% chitosan (26±0.1 mm, 25±0.9 mm, respectively). The maximum zone of inhibition was obtained with 50 mg/mL mangosteen extract (25±0.3 mm) and 400 mg/mL Triphala extract (24±0.5 mm) for *E. faecalis*. For *C. albicans*, the maximum zone was obtained with 500 mg/mL mangosteen (23±0.2 mm) and 100 mg/mL of Triphala extract (23±0.7 mm). No zone of inhibition was seen with the negative solvent control group. This maximum zone of inhibition concentration was used as the MIC for evaluating effectiveness against biofilm. For dual-species, the maximum effective tested doses obtained for mangosteen and Triphala were considered for further experiments.

### SEM analysis

**Figure 2.**
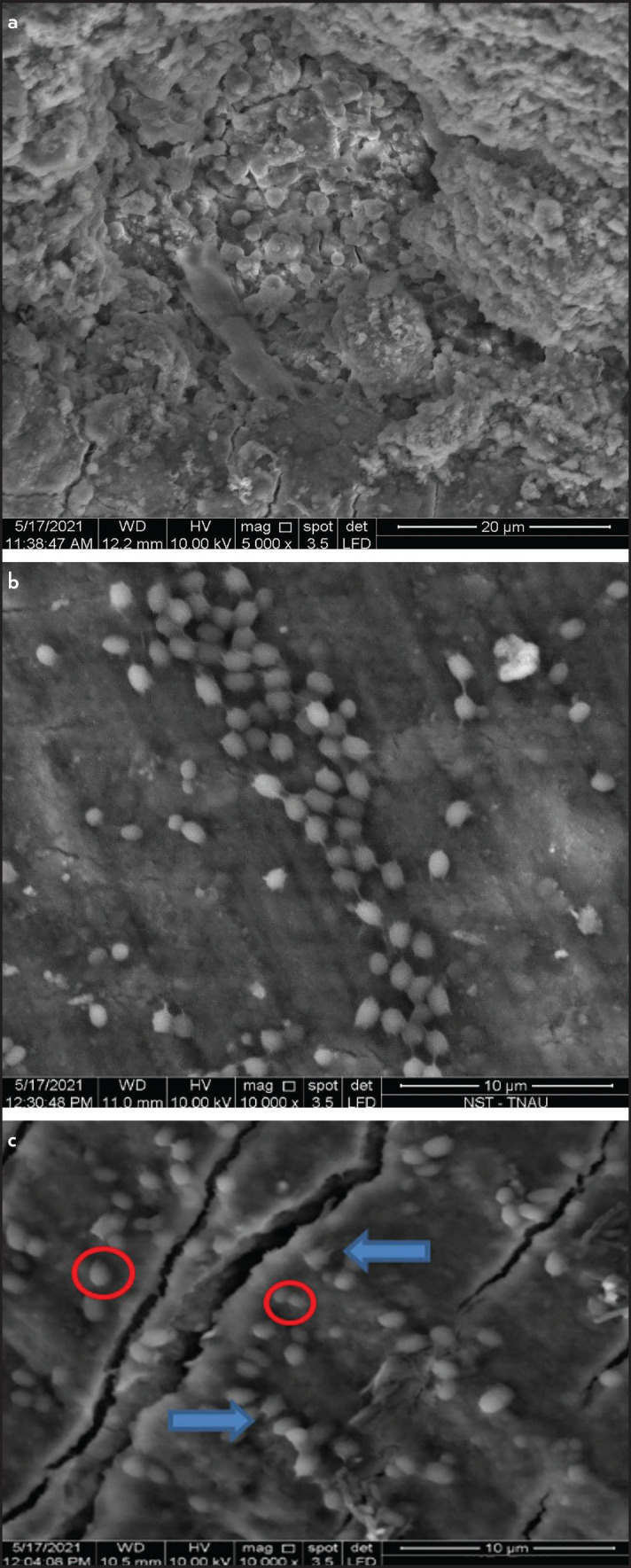
Scanning electron microscopy images -mono- (a, b) and dual-species (c) biofilms of Enterococcus faecalis and Candida albicans formed on the surface of root dentin surface. The arrow indicates Candida albicans and the circle indicates Enterococcus faecalis. Original magnification, ×5000 (a) and x10000 (b, c)

Scanning electron microscopy images of mono- (A, B) and dual-species (C) biofilms of *E. faecalis* ATTC 29212 and *C. albicans* ATCC 24433 formed on the surface of the root dentine are shown in Figure 2.

### Biofilm formation and extracellular polysaccharide production in the culture medium

In the presence of medicaments, significant inhibition of biofilm formation and extracellular polysaccharide production were noted in all tested medicament groups compared to the distilled water control group (Table 3). Among the tested medicaments, better inhibition of biofilm formation by *E. faecalis* and *C. albicans* was noted in the chlorhexidine and chitosan groups compared to mangosteen and Triphala. Better inhibition of dual-species interkingdom biofilm formation was also noted in chlorhexidine, which was followed by chitosan.

**TABLE 3.** Biofilm formation and exopolysaccharide production inhibition

Treatment	Biofilm formation (CV-OD595 values)			Exopolysaccharide production (µg/mL)	
	*E. faecalis*	*C. albicans*	Dual-species	*E. faecalis*	*C. albicans*
Distilled water	1.7±0.01^e^	1.6±0.01^e^	2.0±0.00^e^	2.6±0.08^e^	2.9±0.10^e^
Chlorhexidine	0.0±0.00^a^	0.0±0.00^a^	0.0±0.00^a^	0.0^a^	0.0^a^
Chitosan	0.1±0.00^b^	0.4±0.05^b^	0.4±0.00^b^	1.6±0.07^b^	1.2±0.29^b^
Mangosteen	0.3±0.00^c^	0.8±0.00^c^	1.7±0.00^c^	2.0±0.09^c^	1.9±0.05^c^
Triphala	1.1±0.01^d^	1.0±0.00^d^	1.5±0.00^d^	2.1±0.16^d^	2.1±0.07^d^

CV-OD595: Crystal violet-optical density at 595 nm. Values in each column are the mean of three replication of ±SD (standard deviation). Values with different superscript letters indicate significant differences (P<0.05)

### CFU analysis of tooth substrate biofilm analysis

CFUs were converted into log10 to normalise the data and perform statistical analysis. There was a significant reduction of viable bacteria in all medicament groups compared to the control (P>0.05). All the tested medicament groups showed a significant reduction in mono-species biofilm on both days. The chlorhexidine group showed maximum log reduction of the organisms, followed by the chitosan group, which showed a similar log reduction (P>0.05) for both mono- and dual-species biofilms. On both days, the CFU/mL in mangosteen and Triphala groups for dual-species did not significantly reduce CFUs (P<0.05) compared to the chlorhexidine and chitosan groups. Compared to the first day, all the tested medicaments’ groups (chlorhexidine, chitosan, mangosteen, and Triphala) showed a reduction in count on the fifth day. The results of the analyses mentioned above are shown in Table 4.

**TABLE 4.** Determination population of microorganisms in samples from root canals at two different time intervals

	1^st^ day				5^th^ day			
			Dual-species				Dual-species	
Group	*E. faecalis* Mono-species	*C. albicans* Mono-species	*E. faecalis*	*C. albicans*	*E. faecalis* Mono-species	*C. albicans* Mono-species	*E. faecalis*	*C. albicans*
Distilled water	6.7±0.22^a^	6.5±0.39^a^	5.11±0.26^a^	5.1±0.43^a^	7.45±0.41^a^	6.6±0.47^a^	5.54±0.28^a^	5.88±0.47^a^
Chlorhexidine	1.92±0.36^b^	2.13±0.22^b^	2.03±0.18^b^	2.25±0.24^b^	0.99±0.15^b^	1.05±0.26^b^	0.93±0.14^b^	1.40±0.25^b^
Chitosan	1.98±0.35^b^	2.2±0.21^b^	2.07±0.23^b^	2.36±0.24^b^	1.04±0.15^b^	1.13±0.25^b^	1.23±0.21^b^	1.54±0.24^b^
Mangosteen	1.99±0.36^b^	2.42±0.21^b^	2.57±0.21^c^	3.08±0.15^c^	1.27±0.24^b^	1.90±0.25^b^	2.28±0.24^c^	2.70±0.18^c^
Triphala	2.06±0.34^b^	2.5±0.21^b^	3.87±0.29^c^	3.14±0.29^c^	1.41±0.37^b^	1.96±0.25^b^	4.48±0.36^c^	3.01±0.19^c^

Values with different superscript alphabets indicate a significant difference (P>0.05)

## DISCUSSION

The objective of this study was to compare the antimicrobial effect of natural extracts against *E. faecalis* and *C. albicans*. *Garcinia mangostana*, commonly called mangosteen fruit, was used as an antibacterial agent. Its pericarp is rich in compounds called xanthones, which includes compounds like α-mangostin, β-mangostin, γ-mangostin, garcinone B, and garcinone E. Several studies showed that xanthone has antioxidant, anti-inflammatory, anti-allergy, antibacterial, anticancer, and antifungal effects. In addition, flavonoids from mangosteen pericarp possess antibiofilm activity against *E. faecalis* (14). Triphala contains tannins, quinones, flavones, flavonoids, flavonols phenols, glycosides, gallic acid, and vitamin C, which are responsible for its strong antioxidant activity apart from its antimicrobial effect. The GC-MS analysis of mangosteen extract showed the presence of bioactive compounds, like 2-Methoxy-4-vinylphenol, Tetratetracontane, n-Hexadecanoic acid, aR-Turmerone, 7, 8-Epoxylanostan-11-ol, 3-acetoxy, and Diisooctyl phthalate, which have potential antimicrobial activity (15-17). Similarly, the GC-MS analysis of Triphala extract showed the presence of bioactive compounds, like 9, 12-Octadecadienoic acid (Z, Z)-, Lupeol, n-Hexadecanoic acid, and 9-Octadecenoic acid, which have potential antimicrobial activity (18, 19).

Antimicrobial activity levels of chitosan are related to its degree of deacetylation, and higher antimicrobial activity is observed at lower pH values. Broad-spectrum antimicrobial properties of chitosan are due to the interaction of positively charged chitosan oligomers with the negatively charged microbial cell membranes resulting in leakage of intracellular contents and damage to bacterial cells. 2% chlorhexidine gel and 0.5% chitosan concentrations have shown a better antimicrobial effect; hence, these medicament concentrations were selected in the present study (20-22).

Stojicic et al. (23) investigated the influence of biofilm age on the effectiveness of antimicrobial agents and stated that after 3 weeks of growth, the biofilm bacteria were less susceptible to disinfectants. Previous research proposed that *E. faecalis* and *C. albicans* are usually co-isolated in persistent endodontic infections (24). In the present study, 3-week-old mature biofilms were considered to evaluate and compare the antimicrobial activities of medicaments on mono- and dual-species interkingdom biofilms. In the present study, the formation of mono or dual-species biofilms in the presence of medicaments was evaluated by the crystal violet assay. The analysis of inhibition of *E. faecalis* and *C. albicans* to form biofilms in the presence of medicaments showed that all four test medicaments inhibited biofilm formation, with chlorhexidine and chitosan showing the best results with both mono-species and dual-species. Similar results were also obtained by Costa et al. (25), who stated that chitosan nanoparticles significantly reduced *C. albicans* biofilm formation.

The effect of the medicaments on the mature biofilm that was formed on the root surface was evaluated using viable counts of CFU/mL. The *in vitro* tooth model for assessing CFUs was adapted from Haapasalo and Ørstavik (26). The number of CFUs showed that all the tested medicaments decreased the initial microbial load with a reduction more on day five than on day one. Among the tested medicaments, 2% chlorhexidine showed maximum antimicrobial activity. Regardless of species or biofilm type (mono-species or dual-species), the antimicrobial action of chitosan was comparable to that of chlorhexidine. The result of the present study is similar to the study conducted by Yadav et al. (22), who evaluated the effect of chitosan irrigant against *E. faecalis* and *C. albicans* mono-species and stated chitosan to be effective.

Previous studies have shown that alpha-mangostin exhibited antibacterial activity against *E. faecalis* and also has strong antifungal activity against *C. albicans* (27, 28). The literature showed that Triphala had significant antibacterial activity on mono-species *E. faecalis* biofilm when compared to NaOCl irrigant (10). Choudhary et al. (29) showed that Triphala used as an irrigant had good antimicrobial efficacy against *E. faecalis* and *C. albicans*. A similar result was noted in the present study, where crude extracts of mangosteen and Triphala were used as intracanal medicaments on a mature mono-species biofilm formed on the tooth surface.

In the present study, mangosteen and Triphala had potential action on mono-species biofilm but were not effective on dual-species. This might be due to the complex extra polysaccharide structures seen in the multispecies biofilm model than in mono-species. Besides the protective role of extra polysaccharide structures, microbial interactions in dual-species biofilms may also contribute to resistance enhancement to medicament. In the present study, not much difference was seen in individual microorganism reduction among *E. faecalis* and *C. albicans* in the dual-species model when subjected to medicament. Alshanta et al. (6) stated that *C. albicans* was more susceptible to medicament when co-cultured with *E. faecalis*, which is attributed to the interaction between both organisms. However, this effect will be modified by the conditions of the environment in which the interaction occurs. Also, in work carried out on selected strains of *E. faecalis* and *C. albicans*, it was found that in the dual-species model, *E. faecalis* upregulated several *C. albicans* genes responsible for tissue adhesion and biofilm formation (30). Thus, it is important to keep in mind that further *in vitro* and *in vivo* research using different microbial strains must be conducted to evaluate the antimicrobial effects of using ethanolic extract of mangosteen and Triphala formulations. Also, the efficacy of a natural extract might vary based on the method of extract preparation and solvents employed. In this present study, ethanol extract of natural compounds was used because natural herbs might have a better dissolving capacity in alcohol with better antimicrobial properties (31, 32). The limitations of the present study results that whether they can be extrapolated to the clinical scenario, considering properties such as staining and substantivity of natural extracts, the efficacy of natural medicaments, and the effect on root dentine strength.

Thus, further studies on the multispecies model of clinical isolates, sustained release of medicaments, synergistic effect of natural medicaments when used in combination, and evaluation of clinical trials are to be considered.

## CONCLUSION

Chlorhexidine showed maximum antimicrobial activity, followed by chitosan, on both mono- and dual-species biofilms. Mangosteen and Triphala had good antimicrobial action on monospecies biofilm. Thus natural extracts can be used as a source for the discovery of novel intracanal medicaments from plant sources.
